# Extracellular MicroRNAs in Urologic Malignancies: Chances and Challenges

**DOI:** 10.3390/ijms140714785

**Published:** 2013-07-16

**Authors:** Xiaoyi Huang, Meihua Liang, Rachel Dittmar, Liang Wang

**Affiliations:** 1 Department of Pathology and Cancer Center, Medical College of Wisconsin, Milwaukee, WI 53226, USA; E-Mails: xhuang@mcw.edu(X.H.); rdittmar@mcw.edu(R.D.); 2 Department of Endocrinology, the Second Affiliated Hospital of Harbin Medical University, Harbin 150086, Heilongjiang, China; E-Mail: huangxy01@gmail.com

**Keywords:** urologic cancer, microRNA, extracellular miRNA, microvesicle, exosome

## Abstract

Small noncoding RNAs that are 19–23 nucleotides long, known as microRNAs (miRNAs), are involved in almost all biological mechanisms during carcinogenesis. Recent studies show that miRNAs released from live cells are detectable in body fluids and may be taken up by other cells to confer cell-cell communication. These released miRNAs (here referred to as extracellular miRNAs) are often protected by RNA-binding proteins or embedded inside circulating microvesicles. Due to their relative stability, extracellular miRNAs are believed to be promising candidates as biomarkers for diagnosis and prognosis of disease, or even as therapeutic agents for targeted treatment. In this review, we first describe biogenesis and characteristics of these miRNAs. We then summarize recent publications involving extracellular miRNA profiling studies in three representative urologic cancers, including: prostate cancer, bladder cancer, and renal cell carcinoma. We focus on the diagnostic, prognostic, and therapeutic potential of these miRNAs in biological fluids, such as serum, plasma, and urine. Finally, we discuss advantages and challenges of these miRNAs in clinical applications.

## 1. Introduction

A class of endogenous, single stranded, small noncoding RNAs measuring 19 to 23 nucleotides in length, are known as microRNAs (miRNAs). Like the other members in the small RNA family, miRNAs had been long regarded as transcriptional byproducts. However, current evidence demonstrates that miRNAs have functional implications in physiological, as well as pathological processes. Specifically, these small RNAs are involved in almost all of the known hallmarks of cancinogenesis, including: cell growth, differentiation, proliferation, angiogenesis, apoptosis, and invasion and metastasis [1]. Owing to their relatively strong stability, miRNAs have been extensively studied for their diagnostic and prognostic implications in a variety of human tumors, including urologic cancers [2–5].

Urologic cancers, including cancers of the bladder, kidney, prostate and testicles, are common in Western countries. Among various urologic malignancies, prostate cancer, for example, is the most common non-skin cancer in men, with more than 240,000 cases diagnosed annually in the United States [6,7]. Bladder cancer is the third most common cancer among men and the ninth most common among women in the United States, with an estimated 73,510 new cases and 14,880 deaths in 2012 [7,8]. Despite advances in treatment for these malignancies, urologists still face the challenge of how to improve diagnosis at the early or pre-early stages of disease by developing methods that can detect malignancies, while bypassing the side effects of biopsy and other traditional diagnostic approaches. It is also imperative that active, accurate, and attainable surveillance to optimize the clinical outcome should be enforced during the whole treatment process. In these regards, extracellular miRNAs, either embedded in microvesicles or protected by RNA binding proteins/lipids, are probably capable of providing diagnostic, prognostic, or even therapeutic targets in the battle against urologic tumors.

In this review, recent publications involving extracellular miRNA profiling studies in three representative urologic cancers, prostate cancer, bladder cancer, and renal cell carcinoma, are summarized with focus on their diagnostic, prognostic, and therapeutic roles in biological fluids such as serum, plasma and urine. The advantages and challenges of these miRNAs in clinical applications are discussed. For implications of total cellular miRNAs in urologic tumor tissues, some excellent reviews have been reported elsewhere [5,9,10].

## 2. Biogenesis and Main Functions of Cellular miRNAs

The biogenesis of miRNA is not fully understood yet. In general, cellular miRNA can be divided into two distinct groups based on their origin: endogenous or exogenous. A canonical pathway for miRNA biogenesis follows the Central Dogma, by which miRNA genes are transcribed in the nucleus by RNA polymerase II into primary miRNA hairpins (pri-miRNAs) of more than 500 nucleotides (nt) in length. The pri-miRNAs are cleaved by Drosha/DGCR8 into precursor miRNAs of 65 to 75 nt (pre-miRNAs), and are then transported from the nucleus into the cytoplasm by Exportin 5. In the cytoplasm, pre-miRNAs are further trimmed by Dicer to mature miRNA duplexes in length of 19 to 23 nt [2,11,12]. Canonically generated miRNAs constitute the majority of cellular miRNAs; another source of cellular miRNA is from the extracellular environment. It is now widely accepted that cells may release miRNA-containing complexes into biological fluids such as blood, saliva, urine, and breast milk [9,13]. These complexes include miRNA-binding proteins, such as Argonaute (Ago) [14,15] and high-density lipoprotein (HDL) [16], or miRNA-containing microvesicles [9]. When circulating in blood, they may be taken-up by distant cells, conferring miRNA-mediated cell-cell communication.

Mature miRNAs have significant effects on cellular function through regulation of target mRNAs. The miRNA, along with Ago 2, forms the RNA-induced silencing complex (RISC) and then binds to the 3′-untranslated region (3′UTR) of the target mRNA in a perfect or imperfect pairing manner. If a perfect match occurs between the miRNA and the mRNA, then the target mRNA is directed to endonuclease cleavage; if imperfect pairing results, the target mRNA is directed to translation inhibition. It is estimated that more than 30% of human genes are regulated by about 1000 miRNAs [2,5]. Recently, an AU rich, 3′UTR-induced, RISC-independent miRNA degradation pathway was also suggested [17], which further expanded the possible targets of miRNA. In addition to the expansion of miRNA targets, novel miRNAs have been continuously verified; a total of 2,237 distinct miRNAs are currently listed in miRBase (Release 19, August 2012) [18]. As novel miRNAs continue to be identified, it would not be surprising if every human gene was discovered to have its own regulatory miRNAs.

## 3. Stability and Detectability of Extracellular miRNAs in Biological Fluids

Due to its non-invasive and easily attainable nature, miRNA profiling analysis in biological fluids is an attractive option for biomarker discovery. For a biomarker to be used for diagnostic and prognostic purposes, a minimally invasive method of acquisition is an intrinsic requirement. One common biological fluid for non-invasive collection is urine. Because urine is likely to pass through the malignant lesions in the urologic system before discharge, its miRNA content often reflects the disease status as presented by tumor tissues. Extracellular RNAs in urine and urinary microvesicles may be derived from urologic tumors (such as prostate, bladder and renal cancer), the normal urothelium, the glomerulus, or the renal tubules [19].

Other common biological fluids are serum and plasma that have been reported to contain circulating miRNAs. These extracellular miRNAs have been drawing significantly more research attention over last two decades. Compared to mRNAs, circulating miRNAs are more stable and resistant to physical degradation, such as prolonged storage and freeze/thaw cycles, and to biochemical degradation, such as ribonuclease in serum as well as RNase A *in vitro* [20]. Many mechanisms protect circulating miRNAs in the RNase-rich environment of the blood [21]. For example, Ago1 and Ago2, the co-effector components of the miRNA-induced gene silencing complex, can bind and protect the circulating miRNAs from degradation [14,15]. In addition, high-density lipoprotein (HDL) has also been implicated in transporting endogenous miRNAs to recipient cells via the bloodstream [16]. Despite other protective mechanisms, microvesicles (in particular, exosomes) are recognized as one of the major protective mechanisms in circulation [22,23]. Three different mechanisms have been proposed to explain how the internalization of exosome content occurs [9]. In any case, miRNAs, other small non-coding RNAs, and even mRNAs are packed into the vesicle cargo, transported into the recipient cells, and exert their functions as modulators [9]. This relative stability makes it possible to evaluate circulating miRNAs reproducibly and consistently [24].

Derived from late endosomes and constitutively released by nearly all kinds of cells, exosomes are generally recognized as small intraluminal membrane vesicles ranging from 30 to 100 nm in diameter, at a density between 1.13 and 1.19 g/mL [9,25]. Exosomes are capable of carrying RNA molecules, circulating in blood, and allowing for long distance cell-cell communication [26]. The outer membrane of exosomes provides comprehensive protection by insulating its RNA content from digestive, extracellular environments. The estimated carrying capacity is about 10,000 nucleotides per exosome [9], enough for about 500 miRNAs. In our experience, the isolated exosomes ranged in concentration from 0.88 × 10^8^ to 13.38 × 10^8^ exosomes per 1 mL of stocked serum or plasma (evaluated by NanoSight) [23]. Taken together, miRNA profiles derived from circulating exosomes at least partially reflect the composite collection of miRNAs identified so far. In support of this speculation, over 600 different miRNAs plus nearly 200 putative candidates have been identified from plasma exosomes by our recent RNA sequencing analysis, making up 26% of the total known miRNAs in miRBase [23]. Because tumor cells and tumor-reactive immunocytes actively release exosomes into the bloodstream, circulating exosomes are believed to be a stable RNA source that could be used to identify diagnostic and prognostic markers, or employed as therapeutic agents [9,27–31]. Interestingly, evidence has shown that exosomes in circulation are able to penetrate the glomerular basement membrane and transfer miRNA into urine [32]. Owing to the protective role of exosomes, extracellular miRNAs in urine are prone to be highly stable, making them ideal material for biomarker development.

## 4. Implications of Extracellular miRNAs in Urinary Malignancies

Over the last two decades, accumulating evidence supports the important role of aberrantly expressed miRNAs in urologic malignancies. Since 2008, 25 studies focusing on extracellular miRNA, either in exosomes or in total biological fluid, have been reported in serum, plasma, or urine collected from patients with urologic tumors (Table 1). Regardless of sample type, many miRNAs were found to be differentially expressed in cancer subjects when compared to normal controls. The congruent expression signatures of miRNAs across different studies implicates that there is a general mechanism by which miRNAs modulate carcinogenesis in urologic cancers. This section provides an update regarding the most recent advances in diagnostic, and prognostic, and therapeutic potentials of extracellular miRNAs in prostate, bladder and renal cancers. Due to their low rates of incidence, extracellular miRNAs in other urologic cancers are rarely reported. In this review, extracellular miRNAs are defined as any circulating miRNAs isolated from serum, plasma, secretory microvesicles, or urinary supernatant. The miRNAs isolated from urine sediments are also included in this review for comparison purposes.

### 4.1. Diagnostic and Prognostic Implications

Among urologic tumor-associated extracellular miRNAs, miR-141 and miR-375 are the most consistently reported to be associated with high-risk prostate cancers, such as cancers with a high Gleason score or lymph-node metastasis [34]. Both miRNAs in blood were reported to discriminate metastatic castration resistant prostate cancer from low-risk, localized prostate cancer patients [40]. Their high expressions were also correlated with a reduced relapse-free survival [39]. Serum levels of miR-141 were able to detect cancer in patients with 60% sensitivity and 100% specificity [33]. By isolating exosomes and microvesicles from blood, and using the embedded RNA as starting material, Bryant *et al.* further demonstrated significant up-regulations of miR-107, miR-141, miR-375, and miR-574-3p in metastatic prostate cancer patients compared to non-recurrent cancer patients [37]. The up-regulation of miR-141 in prostate cancer patients was also shown in cellular RNA prepared from urine sediments [37], as well as from tissue samples [55], suggesting the diagnostic and prognostic potentials of miR-141 for prostate cancer. Thus far, the study conducted by Bryant [37] is the only report regarding extracellular exosomal RNAs for biomarker discovery in prostate cancer. At this time, similar studies using RNAs derived from microvesicles in the biological fluid have not been reported in renal or bladder cancer.

For total plasma/serum RNAs, miR-21 and miR-221 are reported to discriminate prostate cancer patients with intermediate risk from those with low risk at a sensitivity of 38.1% and a specificity of 94.2% [43]. Another study using serum revealed four down-regulated miRNAs (miR-24, miR-26b, miR-30c, and miR-223) and six up-regulated miRNAs (miR-20b, miR-93, miR-106a, miR-874, miR-1207-5p, miR-1274a) in prostate cancer patients. Among these, three (miR-24, miR-93, miR-106a) showed consistent expression changes when metastatic patients were compared to healthy controls [36]. Other than single miRNA, Chen *et al.* developed a panel of five miRNAs (miR-30c, miR-622, miR-1285, miR-let-7c, and miR-let-7e) to discriminate prostate cancer from normal individuals and benign prostatic hyperplasia (BPH) with 61% to 90% sensitivity and 57% to 75% specificity [35]. Similarly, a four miRNA panel (miR-16, miR-26a, miR-195, and miR-let-7i) was reported to efficiently distinguish prostate cancer from BPH [38].

To investigate diagnostic or prognostic miRNA markers in bladder cancer, most studies use urine samples. A panel of down-regulated miRNAs, including miR-24, was isolated from urine sediments and found in patients with urothelial cell carcinoma [43]. Serum samples have also implicated the miR-24 in a prostate cancer study [33]. In terms of diagnostic potential, miR-1224-3p showed a specificity of 83% and a sensitivity of 77% in bladder cancer detection. A combination of miR-15b, miR-135b, and miR-1224-3p could detect bladder cancer with a sensitivity of 94.1% and a specificity of 51% [45]. Interestingly, the ratio of miR-126 to miR-152 in pellet sediments of urine distinguished bladder cancer at a specificity of 82% and a sensitivity of 72% [46].

Some plasma miRNAs also showed diagnostic potential for bladder cancer [48]. For example, miR-148b, miR-200b, miR-487, miR-541, and miR-566 were up-regulated in the plasma of bladder cancer patients, whereas expression of miR-25, miR-33b, miR-92a/b, and miR-302 was significantly down-regulated. The predictive powers of these miRNAs were at an accuracy of 89% for discriminating bladder cancer from normal controls and 92% for distinguishing invasive bladder cancer from other cases [48].

For renal cell carcinoma (RCC), von Brandenstein *et al.* reported significant up-regulation of miR-15a in the urine of RCC patients compared to patients with other medical conditions [52], suggesting urinary miR-15a as a potential biomarker for RCC. In serum, both miR-378 and miR-451 were shown to be able to distinguish RCC from healthy controls, and that a combination of these two miRNAs improved stratification power with a sensitivity of 81% and a specificity of 83% [51]. Another study showed that serum miR-1233 level was increased in RCC patients with a sensitivity of 77.4% and a specificity of 37.6% [53]. Finally, a fourth study showed that serum miR-210 levels were significantly higher in clear RCC patients than in normal controls with a sensitivity of 81.0% and a specificity of 79.4% in discriminating diagnosis [54]. This accumulating evidence strongly supports that some extracellular miRNAs are excellent candidates for urologic cancer specific biomarker development, while some differential miRNAs may not be good candidate biomarkers. For example, miR-378, along with three other miRNAs, were up-regulated in patient serum (Table 1), but their discrimination power was not sufficient to be able to provide diagnostic information [50].

### 4.2. Implications of miRNA in Cancer Treatment

Prostate cancers can be broadly categorized by their response to androgen deprivation therapy as either androgen dependent (AD) or androgen independent (AI). According to functional studies, some predictive miRNA markers, such as elevated miR-16, miR-141, and miR-let-7c in the serum and miR-203 in the urine of urologic cancer patients, hold therapeutic potentials [56–59]. For example, down-regulation of miR-let-7c in prostate cancer specimens is inversely correlated with androgen receptor (AR) expression [58]. Through the Myc signaling pathway, miR-let-7 suppressed expression of ARs, inhibited AR activity, and therefore reduced proliferation of prostate cancer cells *in vitro* and *in vivo* [58]. The level of miR-141 in serum and plasma was significantly up-regulated in metastatic castration resistant prostate cancer (mCRPC) patients [40,41]. By translational suppression and selective degradation of *Shp* mRNA, miR-141 contributes to AR-regulated transcriptional activity [55]. In addition, it has been reported that overexpression of miR-221/222 in AD cell lines reduced the level of the dihydrotestosterone (DHT)-induced up-regulation of prostate specific antigen (PSA) expression and increased AI growth of LNCaP cells [60]. This *in vivo* and *in vitro* evidence indicates many miRNAs may function as pivotal regulators in the transition from AD to AI prostate cancer.

It has been reported that miR-16 is up-regulated in the plasma of metastatic prostate cancer patients [41], but down-regulated in primary or metastatic prostate cancer tissues [11,61]. Treatment with miR-16, encapsulated by the vehicle atelocollagen, significantly inhibited the growth of prostate tumors in bone, likely by targeting the CDK1 and CDK2 pathways [62]. As a therapeutic agent, *in vivo* and *in vitro* introduction of miR-16 enriched exosomes into prostate cancer cells significantly suppressed the expression level of the target genes of miR-16, and therefore inhibited proliferation of the cancerous cells [63,64]. These important observations underscore the potential prosperity of using exosomal miRNAs to treat urologic cancer. This not only reflects the functional role that miR-16 plays during tumorigenesis, but more importantly demonstrates the *in vivo* therapeutic effects of miRNA via circulation, highlighting the exciting prospect for clinical use of microvesicle-embedded extracellular miRNAs as a cure for prostate cancer.

In bladder cancer, miR-203 was demonstrated to suppress tumors by directly targeting Akt2/Src and Bcl-w. Induced expression of miR-203 led to the down-regulation of Akt2 and Src, as well as a decreased rate of proliferation, and an increase in apoptosis of bladder cancer cells [59,65]. On the contrary to up-regulation in the serum of prostate cancer patients, miR-21 was highly expressed in bladder cancer tissues, and has been shown to promote cell proliferation, as well as chemo resistance in T24 cells through the PI3K-AKT pathway [66]. Overexpression of miR-21 decreased the sensitivity of prostate cancer cells to *docetaxel* through *PDCD4* in PC3 cells, promoting tumor growth [67–69]. This evidence suggests that miR-21 has oncogenetic properties. The other miRNAs listed in Table 1, however, were rarely verified as therapeutic targets *in vitro* or *in vivo*.

Effects of two distinct groups of target genes demonstrate how miRNAs are involved in urologic cancers. In the first group, oncogenes are inhibited by miRNAs, such as miR-2, miR-16, miR-141, and miR-let-7, via direct degradation or translation inhibition. In the second group, tumor suppressors are targeted by oncogenic miRNAs, such as miR-21, via same mechanism. Expression profiles of these miRNAs have been identified and validated by multiple studies employing different sample sets (*i.e.*, whole plasma, serum, or urine; fractions from plasma, serum, or urine), different methods (microarray, real-time qPCR, next generation sequencing), or different normalization standards (*RNU6B, RNU44, RNU48, 5S rRNA*, synthesized *Caenorhabditis elegans* miRNAs, miR-16, and miR-30e). These differentially expressed miRNAs, either alone or in combination, are promising biomarkers that can be used for making earlier diagnoses, more accurate prognoses, better treatment response predictions, and might also make excellent therapeutic agents.

## 5. Major Challenges for Investigating miRNA in Biological Fluids

### 5.1. Conflicts between Different Studies

Concordant miRNA expression patterns in biological fluids of cancer patients demonstrate promising potential for the discovery of disease-associated RNA biomarkers. However, inconsistencies of miRNA signatures are also notable, not only between studies using urinary and plasma samples, but also between studies using plasma and serum in urologic cancers (Table 1). For instance, miR-141 level significantly increased in prostate cancer patients’ serum and plasma in one study [37], decreased in a different study [42], and were undetectable in another study [43]. Similarly, there was no expression difference of miR-141 among serum samples of prostate cancer, bladder cancer, and renal cell carcinoma patients, regardless of the normalization standards used [44]. In addition, expression of miR-16, which was down-regulated in prostate cancer tissues and the first reported potential therapeutic agent [11,61], was not significantly different in serum between cases and controls [33]. In fact, miR-16 itself was even used as an endogenous control for normalization in renal cell carcinoma [51]. Furthermore, miR-155 levels were decreased in both bladder cancer tissues and urinary sediments, but increased in cell free supernatants [47]. Possible explanations for these inconsistencies include the differences in sample source and processing, and in the selection of internal reference controls.

### 5.2. Selection of miRNA Reference Controls for Normalization

While differences in sampling conditions and heterogeneous origins of miRNA may alter the proportion of unique extracellular miRNAs in circulation [70,71], a valid standard for normalization will help minimize these technical influences. Proper normalization will remove systematic bias and experimental variation to ensure detection of true biological differences between samples. To precisely evaluate the therapeutic effects of miRNAs in the course of *in vitro* and *in vivo* experiments, or in clinical trials, reliable reference controls for normalization are especially important.

In urine, creatinine levels seem to hold some promising possibilities to normalize miRNAs [72]. However, this option for normalization may raise questions about the eligibility of creatinine because it is apparently not the same biomolecule type as miRNAs, and therefore may react differently during the same biological event. In serum, *RNU43* seems to be the most stable reference control for circulating RNAs [44]. *RNU43* may be used as a single reference, or combined with *RNU1-4* as reference controls for stronger stability in urologic cancers. Other urologic cancer studies have used miR-16 and miR-30e as blood-based endogenous controls [41,51]. Although lacking a valid standard, normalization by input volume is prevalently applied when measuring extracellular miRNAs in many studies [70,72] where some synthetic miRNAs have also been adopted as exogenous spike-in controls for normalization. *C. elegans* miRNAs, such as cel-miR-39, cel-miR-43, cel-miR-54, and cel-miR-238, are generally used as reference controls for assessing human miRNA expression levels, especially in circulating RNA studies (Table 1) [37,73,74]. However, the same input volume of the biological fluid does not guarantee an equal amount of miRNAs in the specimen. In addition, exogenous spiked-in miRNAs are merely capable of correcting technical variations, such as different efficiency of RNA isolation and reverse transcription PCR. Therefore, intrinsic biological variations among different study subjects cannot be effectively normalized by this strategy. Taken together, criteria that define a valid extracellular miRNA standard must be the same biotypes as the targets, highly conserved across species, highly abundant, perfectly compatible to different techniques, and more importantly, stably and universally expressed irrespective of biological variance, medical conditions and treatments. Accordingly, an ideal extracellular miRNA standard may emerge from the miRNA repertoire existing in biological fluids, as seen *β-actin* and *GAPDH* for endogenous control of cellular mRNA.

## 6. Conclusions and Future Prospects

The extracellular miRNAs have shown a great potential as diagnostic and prognostic biomarkers in urologic cancers. Due to differences in study subjects and methodologies, some of these studies also yield conflicting data and outcomes. It is important to standardize patient recruitment, sample collection, RNA isolation and quantification. There is urgent need to identify an endogenous reference miRNA for extracellular miRNA normalization. To date an appropriate standard by which miRNA levels can be normalized precisely and efficiently in both basic science and clinical research is on the horizon. The universal adoption of validated miRNA standards would enhance the future prospects of extracellular miRNAs in diagnosis, prognosis, surveillance, or in therapeutic application. The assays based on the extracellular miRNA expression signatures may prove useful as a noninvasive test to guide a physician’s clinical decision on comprehensive management of patients with urologic cancers.

## Figures and Tables

**Table 1:** Expression signatures of extracellular miRNAs in prostate, bladder and renal cancers.

miRNAs	Case/control (size)	Sample	Clinical implications	Regulation	Standard	References
**Prostate cancer (PC)**						
miR-141	metastatic PC(25)/NC(25)	Serum	Diagnosis of advanced prostate cancer	Up	cel-miR-39, cel-miR-54, and cel-miR-238	[33]
miRNA-375 and miRNA-141	metastasized PC(10)/localized PC(59)/high-risk tumors(48)/intermediate risk tumors(23)	Serum	Diagnosis of advanced prostate cancer	Up	cel-miR-39, cel-miR-54, cel-miR-238	[34]
miR-let-7e, miR-let-7c, and miR-30c	PC(105)/BPH(61)/NC(54)	Plasma	Diagnosis of prostate cancer	Down	*RNU6B*	[35]
miR-622 and miR-1285				Up		
miR-24, miR-26b, miR-30c, and miR-223	PC(36)/NC(12)	Serum	Diagnosis of prostate cancer and prognosis of disease progression	Down	Global median normalization	[36]
miR-874, miR-1274a, miR-1207-5p, miR-93, and miR-106a				Up		
miR-107, miR-141, miR-375, and miR-574-3p	PC(78)/NC(28)	Plasma MVs	miR-375 and miR-141: metastasis; miR-107, miR-574-3p: diagnosis	Up	cel-miR-39	[37]
	metastatic PC (47)/non-recurrent disease (72)	Serum MVs			cel-miR-39	
	PC(118)/NC(17)	Urine cell pellets			*RNU44* and *RNU48*	
miR-26a, miR-195, and miR-let-7i	Localized PC(37)/metastatic PC(8)/BPH(18)/NC(20)	Serum	miR-26a for the discrimination of PCA and BPH patients	Up	cel-miR-39	[38]
miR-141, miR-298, miR-346, and miR-375	mCRPC(25)/NC(25)	Serum	Prediction of clinical outcome	Up	cel-miR-39	[39]
miR-375, miR-378a-5p, and miR-141	Based on the D’Amico risk classification criteria mCRPC(26)/localized, low-risk (28)/high-risk (30)	Serum	Diagnosis of metastatic CRPC from low-risk, localized prostate cancer	Up	*RNU6B*, cel-miR-39, cel-miR-54, and cel-miR-238	[40]
miR-409-3p				Down		
miR-16, miR-126, miR-141, miR-151-3p, and miR-375	mCRPC(25)/localized PC(25)	Plasma	Diagnosis of metastatic CRPC from localized prostate cancer	Up	miR-30e	[41]
miR-141	PC (21)	Plasma	Prediction of clinical course and response to therapy	Down	NA	[42]
miR-21 and miR-221	Cancer of the Prostate Risk Assessment Score: Low risk(52)/intermediate risk(21)/high risk(9)	Plasma	Diagnosis of intermediate risk from low risk CAPRA scores, but not eligible to predict PCa aggressiveness	Up	Chemically synthesized RNA oligonucleotides	[43]
miR-21	PC (24)/NC(48)	Serum	Not significant for diagnosis		cel-miR-39, *RNU43*, *RNU1-4*	[44]
**Bladder cancer (BC)**						
miRs-15a/b, miR-24-1, miR-27b, miR-100, miR-135b, miR-203, miR-212, miR-328, miR-1224	Urothelial cell carcinoma patients (85)/NC(53)	Urine cell pellets	Diagnosis of UCC	Down	*RNU48*	[45]
ratio of miR-126:miR-152	low-grade BC(9)/with high-grade BC(9)/urinary tract infections(9)/NC(9)	Urine	Diagnosis of BC	Down	*RNU6B*	[46]
miR-155, miR-192, miR-200 family, and miR-205	BC(51)/NC(24)	Urine sediment and supernatant	Diagnosis of BC	Down	*RNU48*	[47]
miR-25, miR-33b, miR-92, and miR-302	BC(20)/NC(18)	Plasma	Diagnosis of invasive BC	Up	*RNU6B*	[48]
miR-141, miR-148b, miR-200b, miR-487, miR-541, miR-566,	BC(20)/NC(18)	Plasma	Diagnosis of BC and invasive BC	Down?	*RNU6B*	[48]
and miR-639	BC(148)/patients with non-malignant urological disease(115)	Serum	Not significant for diagnosis	Up	cel-miR-39	[49]
miR-21	BC (24)/NC(48)	Serum	Not significant for diagnosis		cel-miR-39, *RNU43*, *RNU1-4*	[44]
**Renal cell carcinoma (RCC)**						
miR-21	RCC(24)/NC(48)	Serum	Not significant for diagnosis		cel-miR-39, *RNU43*, *RNU1-4*	[44]
miR-26a-2-3p, miR-191, miR-337-3p, and miR-378	RCC(142)/Benign renal tumor(14)/NC(134)	Serum	Not significant for diagnosis	Up	cel-miR-39	[50]
miR-378	RCC(105)/NC(47)	Serum	Diagnosis of RCC	Up	miR-16	[51]
miR-451				Down		
miR-15a	RCC(10)/RCC regressive(9)/oncocytoma(5)/urothelial carcinoma(5)/inflamation(1)/other malignancies(15)	Urine	Diagnosis of RCC	Up	5S rRNA	[52]
miR-1233	RCC(123)/NC(129)/Benign renal tumor(13)	Serum	Diagnosis of RCC	Up	cel-miR-39	[53]
miR-210	Clear RCC(68)/NC(42)/	Serum	Diagnosis of RCC	Up	5s rRNA	[54]

mCRPC: metastatic castration resistant prostate cancer; BPH: benign prostatic hyperplasia; NC: normal control; MV: microvesicle.
